# Effect of Excitation Wavelength on Optical Performances of Quantum-Dot-Converted Light-Emitting Diode

**DOI:** 10.3390/nano9081100

**Published:** 2019-08-01

**Authors:** Caiman Yan, Xuewei Du, Jiasheng Li, Xinrui Ding, Zongtao Li, Yong Tang

**Affiliations:** 1 Key Laboratory of Surface Functional Structure Manufacturing of Guangdong High Education Institutes, South China University of Technology, Guangzhou 510641, China; 2 Foshan Nationstar Optoelectronics Company, Ltd., Foshan 528000, China

**Keywords:** colorimetry, excitation wavelength, light-emitting diode, quantum dots

## Abstract

Light-emitting diode (LED) combined with quantum dots (QDs) is an important candidate for next-generation high-quality semiconductor devices. However, the effect of the excitation wavelength on their optical performance has not been fully explored. In this study, green and red QDs are applied to LEDs of different excitation wavelengths from 365 to 455 nm. The blue light is recommended for exciting QDs from the perspective of energy utilization. However, QD LEDs excited at 365 nm have unique advantages in eliminating the original peaks from the LED chip. Moreover, the green or red light excited by ultraviolet light has an advantage in colorimetry. Even for the 455 nm LED with the highest QD concentration at 7.0 wt%, the color quality could not compete with the 365 nm LED with the lowest QD concentration at 0.2 wt%. A 117.5% (NTSC1953) color gamut could be obtained by the 365 nm-excited RGB system, which is 32.6% higher than by the 455 nm-excited solution, and this can help expand the color gamut of LED devices. Consequently, this study provides an understanding of the properties of QD-converted LEDs under different wavelength excitations, and offers a general guide to selecting a pumping source for QDs.

## 1. Introduction

Light-emitting diodes (LEDs) are fundamental devices for backlights and displays [1,2]. Nowadays, the common base pixel is composed of red, green, and blue (RGB) tricolor in both RGB backlights and direct display technology. Three different-color epitaxial chips are mainly applied in an RGB pixel: Blue, green, and red LED chips [3]. However, this solution cannot avoid the following problems: First, the blue, green, and red LEDs have different turn-on voltage characteristics, resulting in voltage balance problems in the circuit design [4]; second, owing to process limitations, the optical efficiency and production yield of red LEDs cannot compare with blue LEDs [5], resulting in high technology thresholds and increased costs; third, it is difficult to further improve the color quality in the current common RGB pixel because of the epitaxial material property limitation.

In order to solve the above difficulties, there are two advanced technologies under discussion. The first advanced technology is using only one LED chip, such as a blue or ultraviolet chip, to solve the voltage mismatch problem [6,7]. For the blue chip solution, the blue light is from a blue LED chip; the green and red light are obtained from the blue light to excite the green and red fluorescent materials. For the ultraviolet chip solution, the blue, green, and red light are all obtained from the ultraviolet-light-excitation fluorescent material [8]. Another technique is to maintain the mature commercial blue chip as blue light and introduce an ultraviolet chip to excite green and red fluorescent materials in order to achieve green and red light. Among these strategies, the core point is that using a short-wavelength light to excite fluorescent materials attains the primary color.

Fluorescent materials are key to improving display performance such as the color gamut. In the past decade, fluorescent materials have evolved from a single YAG:Ce^3+^ phosphor to green and red fluorescent materials such as β-sialon:Eu and CaAlSiN_3_:Eu phosphors [9,10,11,12,13]. However, these fluorescent materials with a half-width wider than 50 nm are not ideal. Further improving the color gamut of LEDs is a hot spot for future commercial competition [14,15]. As a new type of luminescent material, quantum dots (QDs) are quite promising owing to their narrow half-peak width down to 30 nm, wide excitation wavelength, high color purity, and tunable emission [16,17]. 

LED combined with QDs is an important candidate for next-generation high-quality semiconductor devices [18,19]. By taking the advantages of the QD, a green or red LED could restore the natural color to the greatest extent [20]. Shin-Tson Wu et al. applied CdSe QDs for backlight applications, and a 115% color gamut in CIE 1931 was attained [21]. Similar high-quality colors from QD were reported by Shinae Jun [22], Jian Chen [23], and Huang-Yu Lin [24] et al. In order to obtain green and red LEDs, QDs are typically excited by short wavelengths such as blue or ultraviolet (UV) light [24,25]. However, the effect of the excitation wavelength on the optical performance of QD devices has not been systematically studied.

Herein, using CdSe QDs, this study compared the performance of green and red QD LEDs at different excitation wavelengths. The photoluminescence (PL) pattern of QD films was explored. Then, the optical performances of green and red LEDs were compared and analyzed at different excitation wavelengths. It turns out the excitation wavelength of the LED chip has an important impact on the overall device performance.

## 2. Methods

### 2.1. Materials and Equipments

The green and red QDs were purchased from Beijing Beida Jubang Science & Technology Co., Ltd. The PDMS package material was purchased from Dow Corning Corporation, USA. TEM images were taken by a transmission electron microscope (TEM, JEM-2100F, JEOL, Akishima, Japan) operated at an accelerating voltage of 200 kV while dropping the QD solution on the copper network. The PL spectrum was measured by a fluorescence spectrophotometer (RF-6000, Shimadzu, Kyoto, Japan) under 365 nm of excitation. The absorption and transmission spectra were collected by a UV-vis spectrometer with an integrating sphere accessory (TU-1901, Persee, Beijing, China). The electroluminescence (EL) spectra and optical parameters of the LED devices were measured with a calibrated integrating sphere system, a spectrometer (USB2000+, Ocean Optics, Largo, FL, USA), and a power supply (Keithley 2425, Keithley Instruments & Products, Cleveland, OH, USA).

### 2.2. Characterization of Quantum Dot

**Figure 1:**
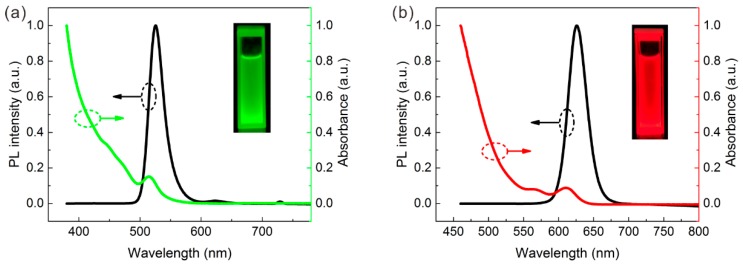
Photoluminescence (PL) and absorbance spectrum of (**a**) GQDs and (**b**) RQDs.

To determine the characteristics of the QDs used in this study, their PL and absorbance spectrum after dispersal in *n*-hexane were measured and are shown in Figure 1. The emission peaks of green QDs (GQDs) and red QDs (RQDs) are located at 525 and 626 nm, while their absorption peaks are at 515 and 610 nm, respectively. This difference between the emission and absorption peaks is attributed to the Stokes shift effect. The half width of the emission peak is 30 nm for GQDs and 28 nm for RQDs. This indicates that the QDs have a narrower half-width than most common fluorescent materials such as YAG:Ce^3+^ phosphor [26,27] and that the color purity can be guaranteed. The inset picture also indicates their excellent color purity. QDs can absorb light of a shorter wavelength than their PL emission peaks, and QDs are more capable of absorbing ultraviolet light than in absorbing blue light such as that at 455 nm.

**Figure 2:**
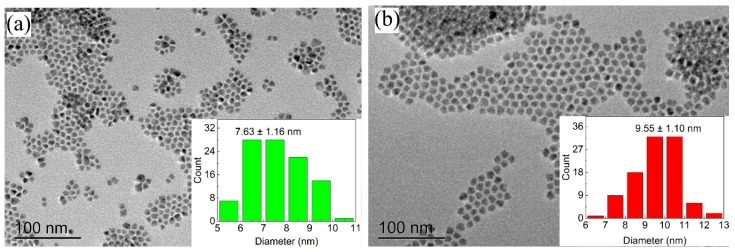
TEM graph of (**a**) GQDs and (**b**) RQDs; inset picture shows particle size distribution of QDs.

From the transmission electron microscope (TEM) graph, all of the QDs show good dispersibility in *n*-hexane. The QDs exhibit a distinct spherical shape, which is formed by the core-shell structure. For these samples, the CdSe acts as a core, and the ZnS acts as a shell. Further, by using ImageJ software to calculate the diameter of 100 QDs in the TEM graph, the particle size distribution is shown in Figure 2. Both particle size distributions show an approximately normal distribution. The average diameter of the GQDs and RQDs is 7.63 ± 1.16 nm and 9.55 ± 1.10 nm, respectively. The particle size of RQDs is larger than that of the GQDs, which is consistent with the reported discovery: the emission peak of CdSe-based QD is related to its particle size, and a larger diameter can result in a longer emission wavelength [28,29].

### 2.3. Fabrication of QD-Based LED with Different Excitation Wavelengths

**Figure 3:**
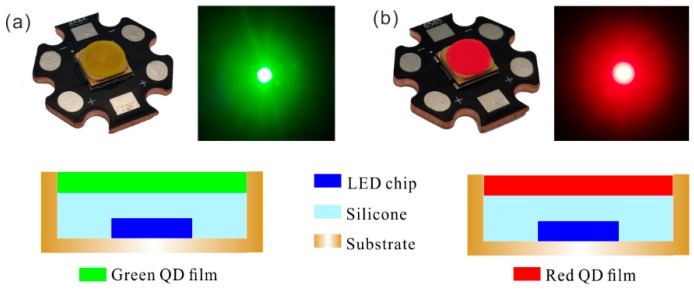
Physical picture and schematic diagram of (**a**) green quantum-dot LED and (**b**) red quantum-dot LED.

To obtain a QD-based LED with different excitation wavelengths for this study, first, commercial LED chips of the same size (45 × 45 mil) with 365, 385, 405, and 455 nm of emission wavelengths were mounted on a copper substrate. The copper substrate serves as a circuit board and a heat sink. Then, the golden wire was connected by ultrasonic welding. For QD applications, the remote excitation to attain green and red light is the basic LED package structure in this study, as shown in Figure 3. Polydimethylsiloxane (PDMS) was selected as the package material. PDMS gel was injected into the cavity and then cured at 100 °C for 1 h.

To prepare QD remote films for LEDs, green CdSe/ZnS and red CdSe/ZnS/ZnS QDs were selected. After dispersal in *n*-hexane, the QDs were mixed with the PDMS at mass concentrations of 0, 0.2, 0.5, 1.2, 3.0, and 7.0 wt%. Then, the film was prepared using a designed mold. The thickness of the film was fixed at 500 μm by a gasket. After 1 h of heating at 100 °C, the mold could be released to obtain QD films. Finally, the QD remote film was assembled to form LED samples with different excitation wavelengths. Green and red QD-converted LEDs (GQD LED and RQD LED) were obtained.

## 3. Results and Discussion

### 3.1. Characterization of QD Film

**Figure 4:**
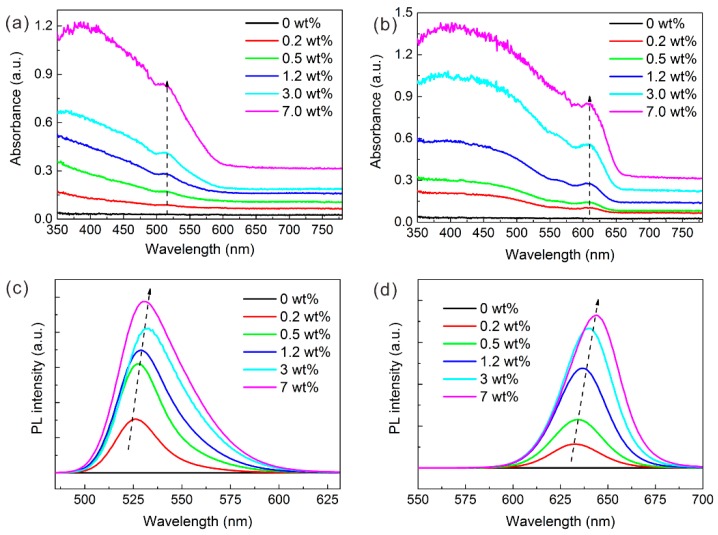
Absorption spectrum of (**a**) GQD and (**b**) RQD film; photoluminescence (PL) spectrum of (**c**) GQDs and (**d**) RQD film.

After the QDs were prepared as QD film, the properties of the QD films were explored. As shown in Figure 4a,b, the absorbance peaks of the GQD and RQD films were located at 512 nm and 610 nm, respectively. These are almost identical to the absorption peak of the QD solution, which means that the film maintains the original luminescence properties of the QDs. The absorbance of both GQD- and RQD-based film increases as the concentration of QDs increases. After adding the QDs, the absorbance below the absorption wavelength increases significantly, and the absorbance of the ultraviolet region is more pronounced than in the blue region. This regular pattern is also reflected in the transmission spectrum, as shown in Figure S1. Thus, with more QDs, more short-wavelength light could be absorbed.

The PL spectra of different QD films excited at 365 nm are shown in Figure 4c,d. The higher the QD concentration, the greater the PL intensity that can be attained. However, a red-shift in the emission peak occurs at a higher QD concentration. For example, at a low concentration of 0.2 wt%, the emission wavelength of GQD film is 526 nm, but at a high concentration of 7 wt%, it is red-shifted to 531 nm, as shown in Figure S2. The reason for the red shift could be the agglomeration and reabsorption of QDs. Agglomeration leads to an increase in the average particle size of the QDs, and the QDs have special properties of reabsorption and reemission [30]. Since the red QD absorption is much stronger than the green QD absorption at 365 nm of excitation, the reabsorption effect is much more significant. Thus, the RQD suffers from a more severe red-shifted effect at high concentrations such as 7.0 wt%.

### 3.2. Light Conversion Optical Model of QD LED

**Figure 5:**
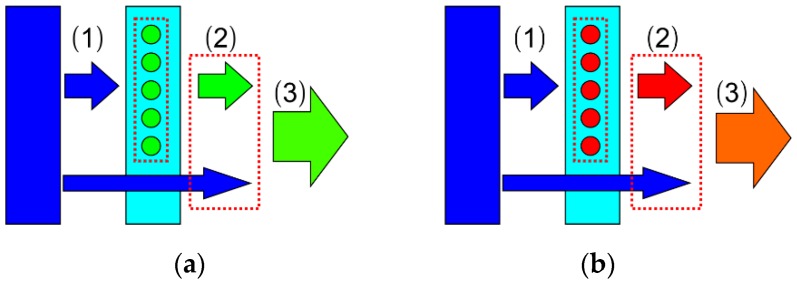
Light conversion optical models of (**a**) GQD LED and (**b**) RQD LED.

With the combination of the LED chip and QD film, the entire device forms a green or red LED, which is the fundamental base of a display pixel. As shown in Figure 5, LED light transformation can be summarized into three stages:

(1) Absorption. The short-wavelength light from the LED chip is absorbed by the QDs. Thus, the QD absorption rate is defined as the ratio of the absorbed short-wavelength light intensity to the initial short-wavelength light intensity. The QD absorption rate can be used to measure the initial peak elimination ability of QDs for short-wavelength light;

(2) Conversion. Then, the QDs can turn the short-wavelength light into their own emission light. For example, the green QD transforms the blue light into green emissions. Thus, the QD conversion efficiency is defined as the ratio of the QD emission intensity to the absorbed short-wavelength light intensity. The QD conversion efficiency reflects the ability to convert short-wavelength light;

(3) Output. After mixing the residual short-wavelength light intensity and the QD emission peak intensity, the LED devices produce new light output. In other words, the final optical output is composed of residual short-wavelength light and QD emissions. Thus, the energy conversion efficiency is defined as the ratio of the final total light intensity to the initial light intensity. This can reveal the energy utilization of the total LED device.

### 3.3. Performance of GQD LED with Different Excitation Wavelengths

**Figure 6:**
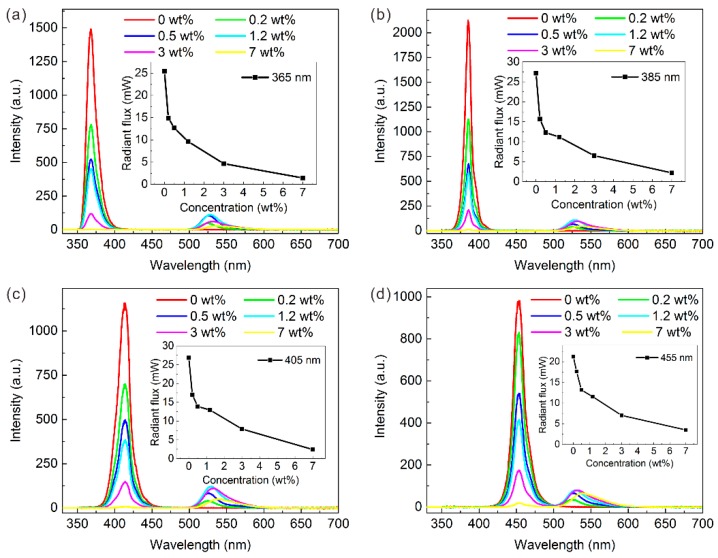
Electroluminescence (EL) spectrum of GQD LED with (**a**) 365 nm, (**b**) 385 nm, (**c**) 405 nm, and (**d**) 455 nm of LED-chip excitation wavelength under 20 mA. Inset graph shows radiation flux vs. GQD concentration.

The EL spectra of different GQD LEDs are explored and shown in Figure 6. At different excitation wavelengths, the following discoveries about QD concentrations are consistent: First, as the concentration of GQD increases, the peak of short-wavelength light decreases while the green peak first rises and then falls. Almost all of the LED samples at different excitation wavelengths show the top green peak intensity at 1.2 wt% of GQD concentration. Second, the green peak emission wavelength shows a red shift with increasing GQD concentration, which is consistent with the PL test of the QD film. Third, the overall radiant flux of the LED device decreased. This confirms the significant energy loss during QD conversion and this situation becomes more serious with high concentration QDs. Therefore, varieties in QD concentration at different excitation wavelengths show similar patterns in the LED spectrum. This means that even if the initial excitation wavelength of the device is changed, the basic shape of the EL spectrum stays the same.

**Figure 7:**
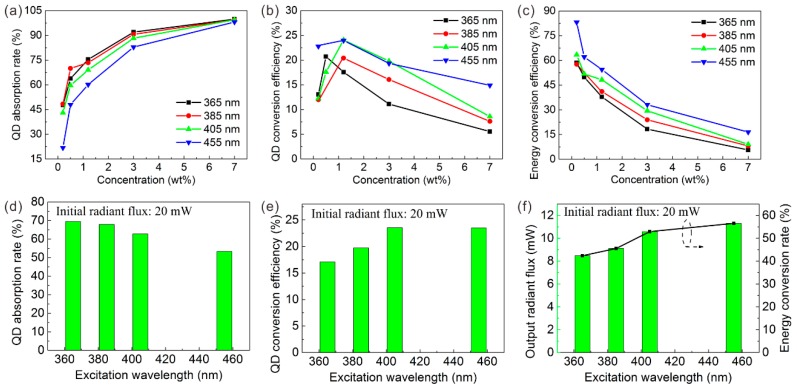
(**a**,**d**) QD absorption rate, (**b**,**e**) QD conversion efficiency, and (**c**,**f**) energy conversion efficiency of GQD LED with different excitation wavelengths.

To further analyze the effects of the excitation wavelength, the optical model described above is introduced here. The first stage is absorption. For the GQD absorption rate, all LEDs with different excitation wavelengths indicate that the higher the QD concentration, the more the short-wavelength light is absorbed, as shown in Figure 7a. However, the GQD absorption rate excited by UV light is much higher than that by blue light with the same QD concentration. Only a GQD concentration of 0.2 wt% can absorb nearly half of the 365 nm original light intensity (47.7%). Further, the initial incident radiant flux of LED chips and the QD concentration (1.2 wt%) were fixed to ensure a single variable of the excitation wavelength. Three initial incident radiant fluxes of the LED chips were studied: 20, 60, and 100 mW. Under a 20 mW incident radiant flux, the GQDs could absorb 69.5% of the 365 nm violet light, while only 53.3% of the 455 nm blue light could be absorbed, as shown in Figure 7d. The ability to absorb short wavelengths of GQD increased by 16.1% under 365 nm of excitation. Thus, a solution that combines the UV light and QDs has advantages in eliminating raw chip peaks.

Then, the absorbed short-wavelength light is converted to green light by GQDs. Among the excitation wavelengths, the QD conversion efficiency of GQD rises first and then drops as the QD concentration increases. Thus, a moderate QD concentration such as 1.2 wt% can maximize the QD conversion efficiency. The QD conversion efficiency tends to increase at longer excitation wavelengths. In other words, the QD conversion efficiency excited by ultraviolet light is lower than that by blue light. Taking the 20 mW incident radiant flux as an example, the QD conversion efficiency is only 17.1% under 365 nm of excitation but increases to 23.5% with 455 nm of excitation, a 6.4% increase. It should be pointed out that the QD conversion efficiency is not ideal; this could be owing to the severe reabsorption characteristics of QDs [31]. In short, typical blue light has an advantage in QD conversion efficiency.

The last part of the optical model is the output. At different excitation wavelengths, the energy conversion efficiency of all LEDs declines as the QD concentration increases. This may be owing to the agglomeration and reabsorption effect of the high QD concentration, which is more likely to produce significant heat [32,33]. Therefore, from the perspective of energy utilization, a low QD concentration is better for LED applications. However, at the same QD concentration, the energy conversion efficiency gradually increases as the excitation wavelength increases. The typical blue 455 nm excited LED has a maximum energy conversion efficiency of 83.1% with a GQD concentration of 0.2 wt%. As shown in Figure 7f, when the incident radiant flux is 20 mW, the output radiant flux of the LED with excitation wavelengths of 365, 385, 405, and 455 nm is 8.5, 9.1, 10.6, and 11.3 mW, respectively. Therefore, the energy conversion efficiency at 455 nm is 14.1% higher than that at 365 nm. Repeated experiments with different incident radiant fluxes such as 60 and 100 mW demonstrate the reliability of the above conclusions, as shown in Figure S3.

**Figure 8:**
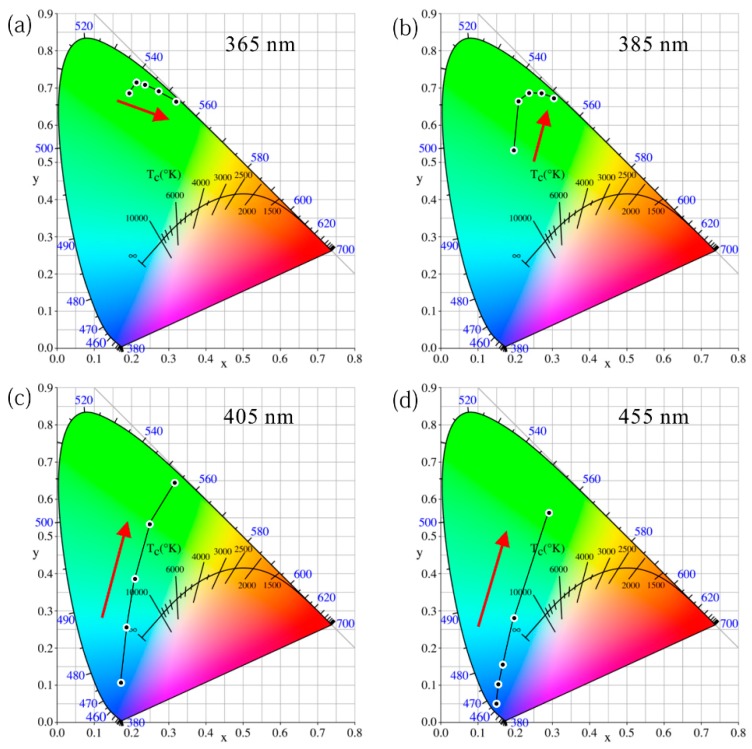
Chromaticity diagram of GQD LED with different QD concentrations under (**a**) 365 nm, (**b**) 385 nm, (**c**) 405 nm, and (**d**) 455 nm of excitation.

To determine the color quality of these different LEDs, their color coordinates are summarized and drawn in the chromaticity diagram in Figure 8. The colorimetric behavior of the GQD LED with different excitations can be divided into two categories: (A) 365 and 385 nm of excitation and (B) 405 and 455 nm of excitation. Under 365 and 385 nm of excitation, the *x* color coordinate keeps increasing, while the *y* color coordinate first increases and then decreases as the GQD concentration increases. This indicates that the green light gradually plays a leading role. The color coordinates only shifted within the green-light area. Interestingly, the LED excited at 365 nm at a low QD concentration of 0.2 wt% emits a relatively high-purity green light. However, 405 and 455 nm-based LEDs show distinct color qualities. Both the *x* and *y* color coordinates keep increasing as the QD concentration increases, which leads to a gradual light shift from the blue area to the green area. Even the green color quality of the 455 nm LED with the highest QD concentration at 7.0 wt% could not compete with the 365 nm LED with the lowest QD concentration at 0.2 wt%. If the LEDs with different excitation wavelengths aim to obtain the same green light color quality, the GQD concentration at 455 nm of excitation should be much higher than that at 365 nm of excitation. Thanks to the excellent color characteristics of the QD itself, the color coordinate of the GQD LED with 365 nm excitation and high GQD concentration at 7.0 wt% is (0.322, 0.663). This green light almost reaches the edge of the chromaticity diagram, which means that ultrawide color gamut performance is possible if this GQD is applied to displays.

**Figure 9:**
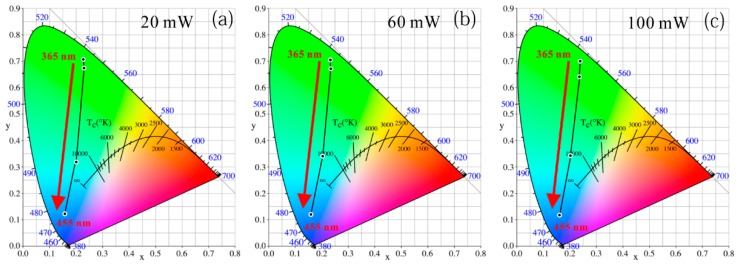
Chromaticity diagram of GQD LED with different excitation wavelengths under equal incident radiant fluxes of (**a**) 20 mW, (**b**) 60 mW, and (**c**) 100 mW.

Even with the same incident light power and the same GQD concentration, the LEDs clearly exhibit different color properties owing to different excitation wavelengths, as shown in Figure 9. The color coordinates of the green LED excited at 365, 385, 405, and 455 nm are (0.231, 0.706), (0.230, 0.674), (0.202, 0.318), and (0.162, 0.123) under a 20 mW incident radiant flux, respectively. Thus, both the *x* and *y* coordinates show a downward trend. In other words, under the excitation of a 365 nm LED chip, the entire GQD device emits high-purity green light. However, under the excitation of the 455-nm LED chip, the device emits blue light. This superiority in color purity excited at 365 nm is mainly owing to two reasons: the GQD LEDs excited at 365 nm have unique advantages in eliminating initial short peaks owing to the stronger absorption ability, and the naked eye is not sensitive to residual 365 nm ultraviolet light. But green light from QD emission is visible and the human eye is quite sensitive to green light. Thus, in terms of color purity, the excitation of QDs by ultraviolet light such as 365 nm can maximize the advantages of the quantum-dot color quality. This UV excitation solution is recommended for display applications.

### 3.4. Performance of RQD LEDs with Different Excitation Wavelengths

**Figure 10:**
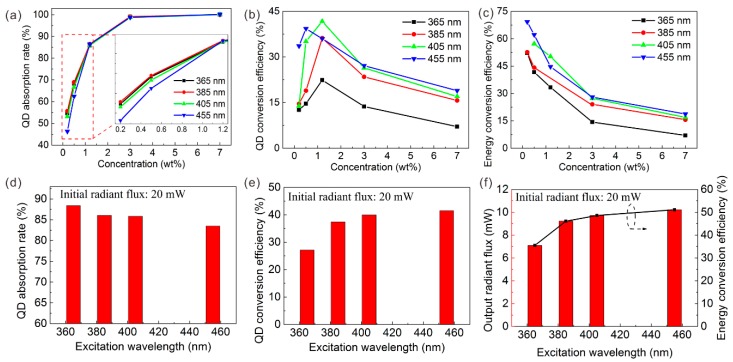
(**a**,**d**) QD absorption rate, (**b**,**e**) QD conversion efficiency, and (**c**,**f**) energy conversion efficiency of GQD LED with different excitation wavelengths.

As for the RQDs, the EL spectrum is also explored and shown in Figure S4. The EL spectra reveal that LEDs based on RQDs exhibit laws similar to those of GQDs. Then, as with the GQD LED, the QD absorption rate, QD conversion efficiency, and energy conversion efficiency of the RQD LED were calculated and are shown in Figure 10. In general, the red LED has a pattern similar to that of the green LED. Unlike green QDs, almost all of the short-wavelength light can be absorbed with only 3.0 wt% concentration RQDs. This reveals that RQDs have a stronger advantage than GQDs in eliminating short peaks. 

Under 20 mW of incident optical power, the RQD absorption rate excited at 365 nm is 5.0% higher than that at 455 nm, but the RQD conversion efficiency and energy conversion efficiency excited at 455 nm are 14.3% and 15.5% higher, respectively, than that at 365 nm. Like the GQD, the blue chip is recommended from the perspective of energy utilization because the maximum energy conversion efficiency is 69.3 wt% from the 455 nm LED. At different incident power such as 60 and 100 mW, the same law is exhibited in Figure S5.

A chromaticity diagram of the RQD LED under different wavelength excitations is also summarized. There is a significant difference in behavior in the color performance between a typical UV 365 nm LED and a blue 455 nm LED. Under 365 nm of excitation, because the original peak from the LED chip is more easily eliminated owing to the stronger absorption ability and the human eye is not sensitive to residual 365 nm ultraviolet light. The main color perceived by the human eye is visible red light, which leads to excellent red-light quality even at a low QD concentration of 0.2 wt%. In addition, the red color quality of the 385 nm excitation is similar to that of 365 nm LED but is slightly closer to the orange color at low concentrations. However, under 455 nm of excitation, the color shifts from blue and purple to red. The LED exhibits significant blue light at low concentrations such as 0.2 wt%. The 405 nm LED shows patterns similar to those of the blue excitation. 

**Figure 11:**
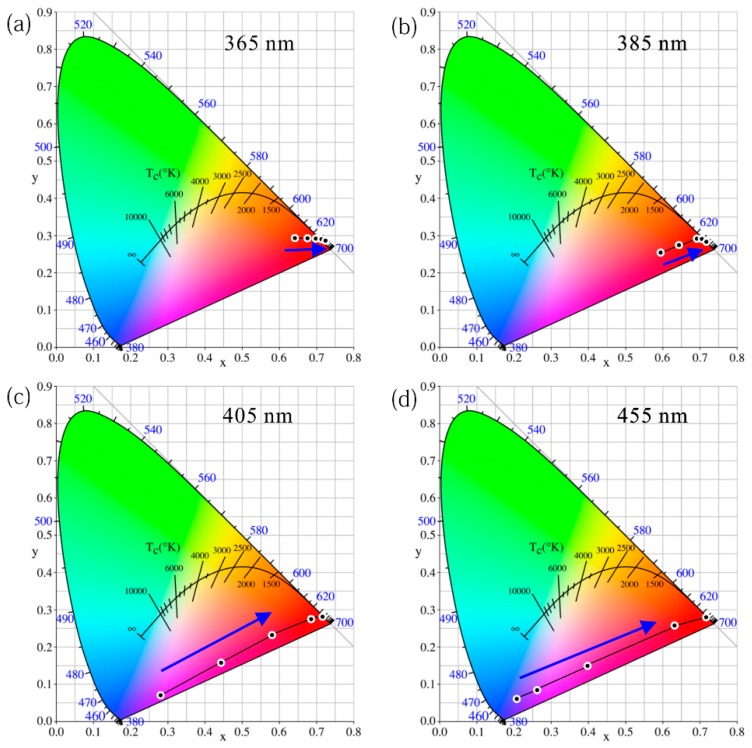
Chromaticity diagram of RQD LED with different QD concentrations under (**a**) 365 nm, (**b**) 385 nm, (**c**) 405 nm, and (**d**) 455 nm of excitation.

Even with the same incident light power and the same RQD concentration (1.2 wt%), the color performance of these LED samples is totally different, as shown in Figure 11. From 365 nm to 455 nm of excitation, the color shifts from red to purple regions, as shown in Figure S6. Taking advantage of the QD, the color coordinates of the 365 nm excitation basically reached the boundaries of the gamut, which means that the red color is highly pure. These experimental results reveal that the combination of UV light with RQDs has unparalleled advantages in colorimetry, which may shine in future display fields. Therefore, the red LED prepared by ultraviolet light to excite RQDs is superior to the blue-light excitation in attaining high-quality red light. This is consistent with the conclusion regarding GQDs.

### 3.5. Comparison of Color Gamut between Blue (455 nm) Excitation and Ultraviolet (365 nm) Excitation Solutions

**Figure 12:**
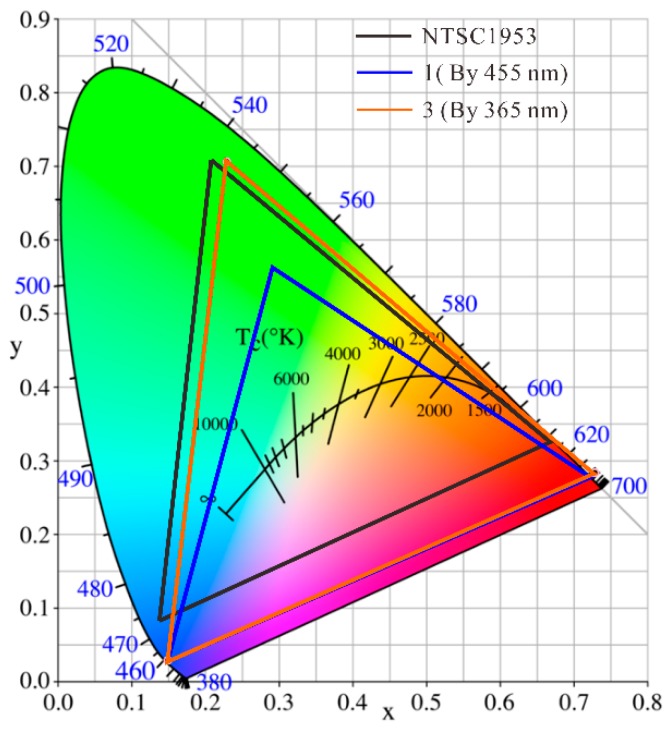
Comparison of color gamut between blue (455-nm) excitation and ultraviolet (365-nm) excitation solutions.

The color gamut is an important parameter when evaluating RGB display systems. The wider the gamut, the higher the fidelity of the colors of nature that can be attained. A comparison of the color gamut for different excitation wavelength solutions is analyzed in Table 1. The blue-color coordinates of all solutions are derived from the blue LED chip, and the green and red color coordinates are obtained from the QD-converted LEDs. With the highest QD concentration at 7.0%, the RGB color gamut excited at 455 nm is only 84.9% of the NTSC1953 standard. Interestingly, the color gamut of 365 nm-excited solution could cover 99.7% of the NTSC1953 standard area even at a low QD concentration (0.2 wt%). Moreover, as shown in Figure 12, the color gamut of a 365-nm-excited RGB system could be high as 117.5% by adjusting the QD concentration properly. This is a 32.6% improvement over the blue-light-excited solution. Thus, the UV-excited quantum-dot solution can achieve a wider color gamut and has great potential for development in quantum-dot applications, especially in the display field.

## 4. Conclusions

In this study, we investigated the effect of the excitation wavelength on the optical performances of QD-converted light-emitting diodes. Sample LEDs with excitation light sources of 365, 385, 405, and 455 nm were prepared, and their optical performances were compared. Under different excitation wavelengths, green and red QD-converted LEDs showed similar patterns. The QD conversion efficiency and energy conversion efficiency increase at longer excitation wavelengths. At an incident radiant flux of 20 mW, the QD conversion efficiency and energy conversion efficiency of the 455 nm-excited GQD LED were 6.4% and 14.1% higher than those of the 365 nm LED. Therefore, blue light is recommended to excite QDs with regard to energy utilization. However, the UV-excited QD LED solution has more advantages than blue-light excitation for color purity. Even for the 455 nm LED with the highest QD concentration at 7.0 wt%, the green color quality could not compete with a 365 nm LED with the lowest QD concentration of 0.2 wt%. This is because QD LEDs excited at 365 nm have unique advantages in eliminating short peaks because of higher absorption in the ultraviolet range of QD. Moreover, the naked eye is not sensitive to the residual 365 nm ultraviolet light. As a result, green and red light excited at 365 nm with a 7.0 wt% QD concentration can approach the boundary of the chromaticity diagram. Taking advantage of the 365 nm-excited solution, a 117.5% color gamut of the RGB system could be obtained compared with the NTSC1953 standard, which is an improvement of 32.6% over the blue-light-excited solution. This research helps understand the variation of quantum-dot-converted LEDs at different excitation wavelengths, and provides basic guidance for the selection of a pumping source for QDs.

## Figures and Tables

**Table 1:** Comparison of color gamut for wavelength solutions with different excitations.

Group	Excitation Wavelength (nm)	GQD (%)	RQD (%)	R(*x, y*)	G(*x, y*)	B(*x, y*)	NTSC1953 Standard (%)
1	455	7.0	7.0	(0.7210, 0.2794)	(0.2935, 0.5624)	(0.1492, 0.0298)	84.9
2	365	0.2	0.2	(0.6485, 0.2931)	(0.1947, 0.6855)	(0.1492, 0.0298)	99.7
3	365	1.2	7.0	(0.7289, 0.2834)	(0.2300, 0.7064)	(0.1492, 0.0299)	117.5

## Supplementary Materials

The following are available online at https://www.mdpi.com/2079-4991/9/8/1100/s1, Figure S1: Transmission spectrum of (a) GQD and (b) RQD film; Figure S2: PL shift phenomenon of (a) GQD film and (c) RQD film; Figure S3: (a,d) GQD absorption rate; (b,e) GQD conversion efficiency; (c,f) output radiant flux and energy conversion efficiency under 60- and 100-mW incident radiant flux; Figure S4: Electroluminescence (EL) spectrum of RQD LED with (a) 365 nm, (b) 385 nm, (c) 405 nm, and (d) 455-nm LED chip excitation wavelength under 20 mA; Figure S5: (a,d) RQD absorption rate; (b,e) RQD conversion efficiency; (c,f) output radiant flux and energy conversion efficiency under 60- and 100-mW incident radiant flux; Figure S6: Chromaticity diagram of RQD LED with different excitation wavelengths under equal incident radiant fluxes such as (a) 20 mW, (b) 60 mW, and (c) 100 mW.
