# Supplementary material for: Effect of Excitation Wavelength on Optical Performances of Quantum-Dot-Converted Light-Emitting Diode
Source: Nanomaterials (Basel). 2019 Aug 1;9(8):1100. doi: 10.3390/nano9081100 (PMC6723292; doi:10.3390/nano9081100)
Supplement: Supplementary file 1 [file nanomaterials-09-01100-s001.zip › nanomaterials-559053-SI.pdf]

nanomaterials

MDPI

Supporting information

Effect of Excitation Wavelength on Optical Performances of Quantum-Dot-Converted Light-Emitting Diode

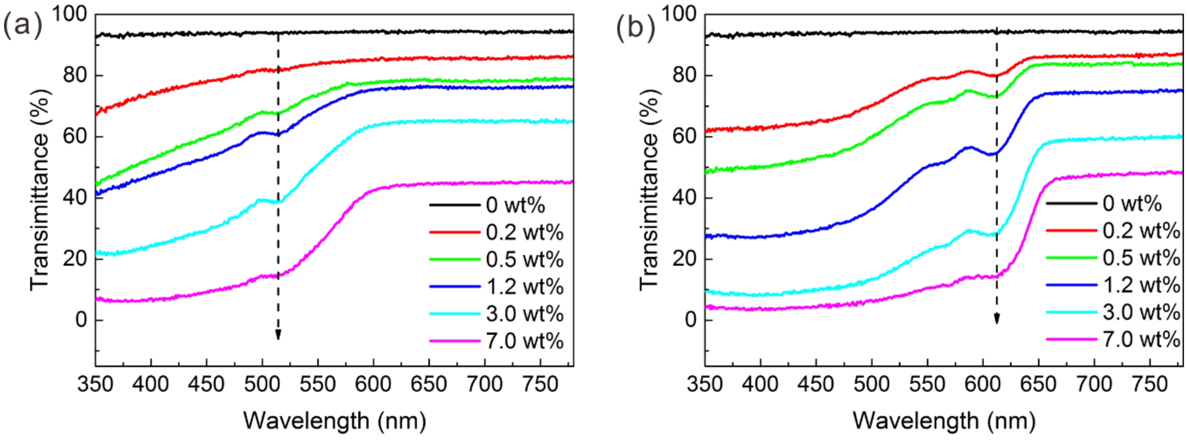

Figure S1. Transmission spectrum of (a) GQD and (b) RQD film.

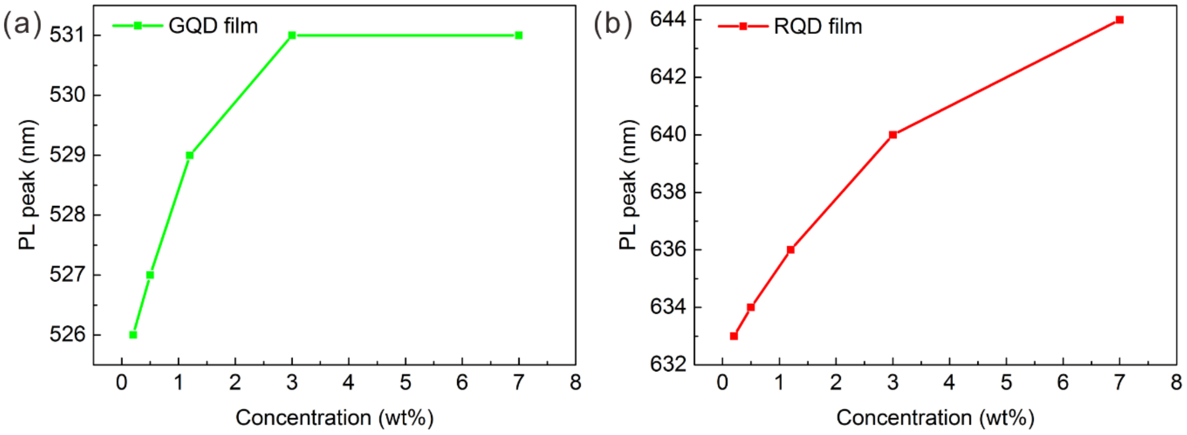

Figure S2. PL shift phenomenon of (a) GQD film and (c) RQD film.

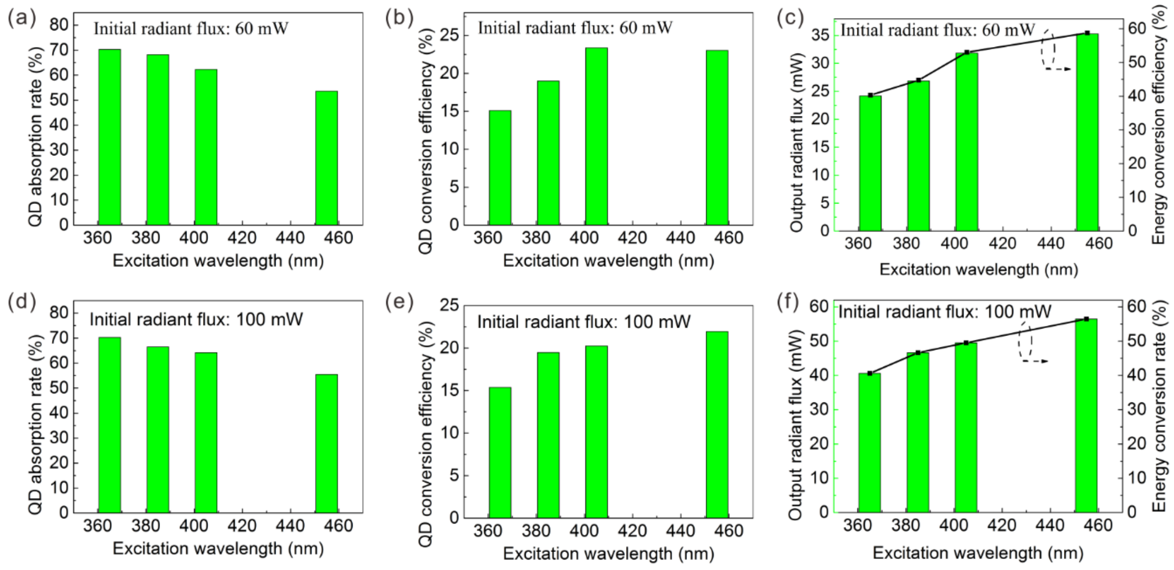

Figure S3. (a,d) GQD absorption rate; **(b,e)** GQD conversion efficiency; **(c,f)** output radiant flux and energy conversion efficiency under 60- and 100-mW incident radiant flux.

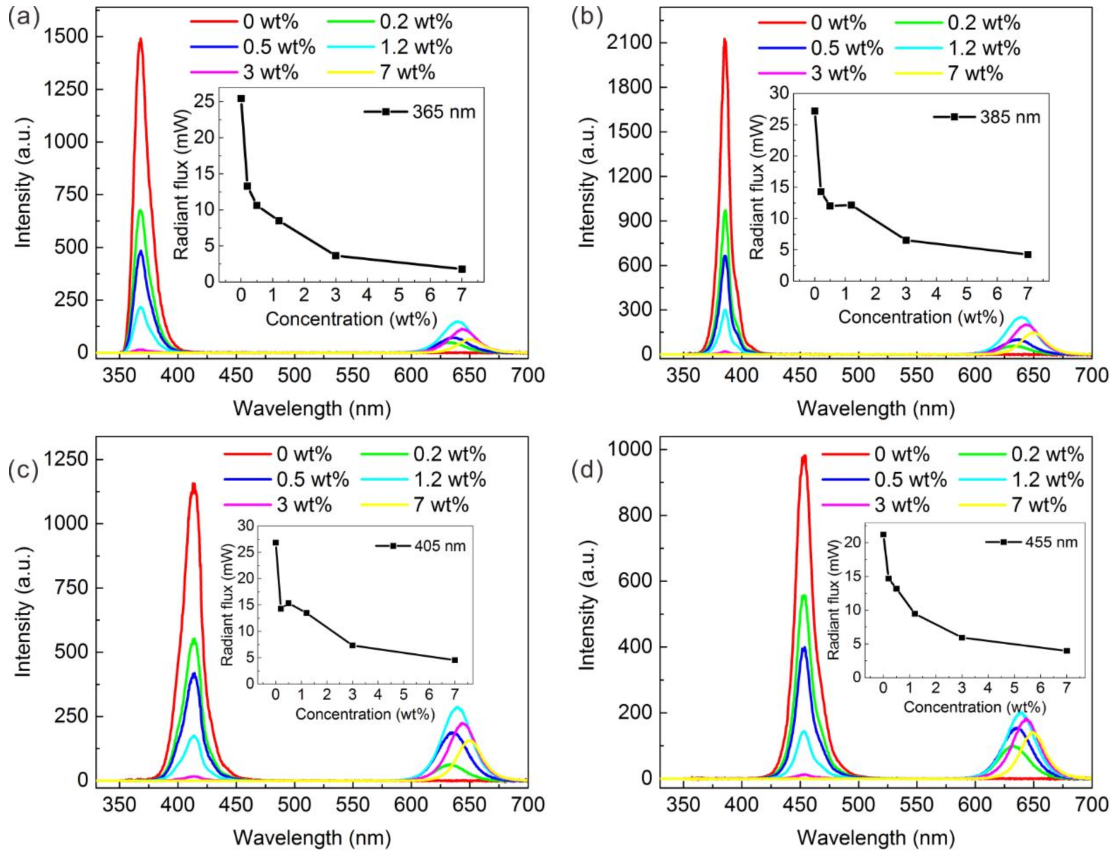

Figure S4. Electroluminescence (EL) spectrum of RQD LED with **(a)** 365 nm, **(b)** 385 nm, **(c)** 405 nm, and **(d)** 455-nm LED chip excitation wavelength under 20 mA. Inset graph shows radiation flux vs. RQD concentration.

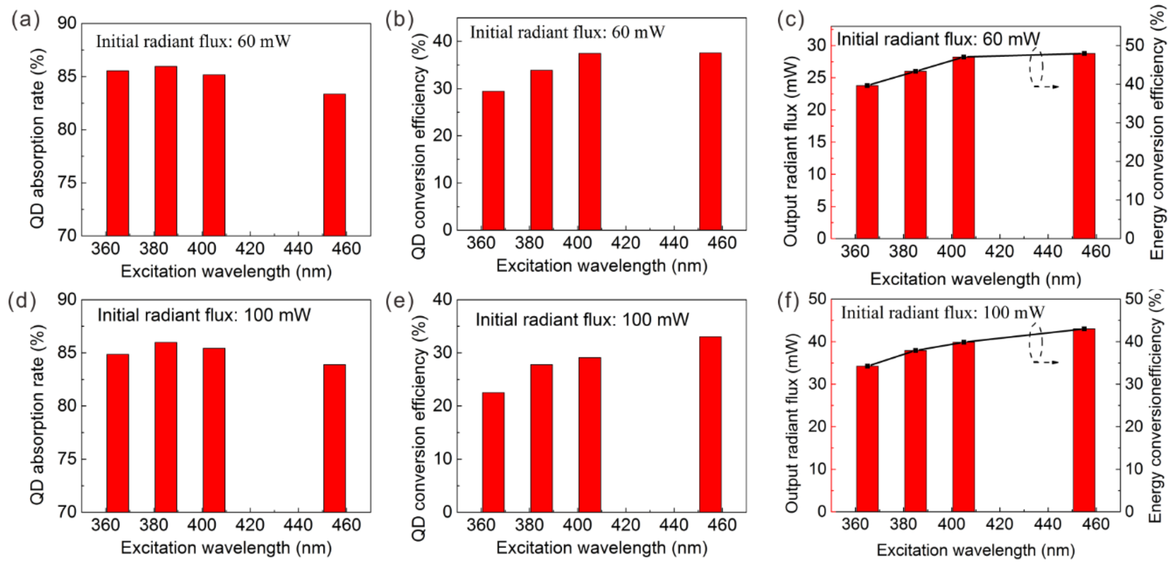

Figure S5. (a)(d) RQD absorption rate; **(b) (e)** RQD conversion efficiency; **(c) (f)** output radiant flux and energy conversion efficiency under 60- and 100-mW incident radiant flux.

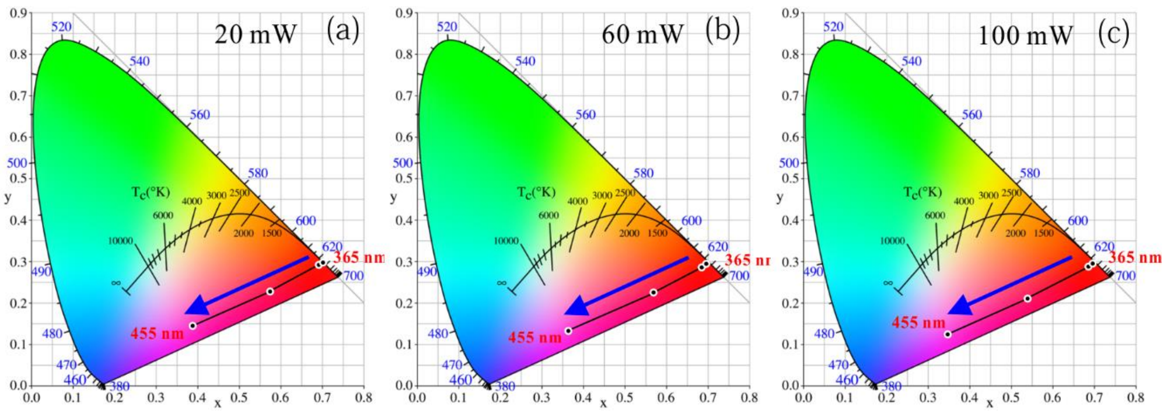

Figure S6. Chromaticity diagram of RQD LED with different excitation wavelengths under equal incident radiant fluxes such as **(a)** 20 mW, **(b)** 60 mW, and **(c)** 100 mW.
